# Predicting the turning points of housing prices by combining the financial model with genetic algorithm

**DOI:** 10.1371/journal.pone.0232478

**Published:** 2020-04-29

**Authors:** Shihai Dong, Yandong Wang, Yanyan Gu, Shiwei Shao, Hui Liu, Shanmei Wu, Mengmeng Li

**Affiliations:** 1 State Key Laboratory of Information Engineering in Surveying, Mapping and Remote Sensing, Wuhan University, Wuhan, China; 2 Collaborative Innovation Center of Geospatial Technology, Wuhan, China; 3 Faculty of Geomatics, East China University of Technology, Nanchang, China; 4 Wuhan Natural Resources and Planning Information Center, Wuhan, China; University of Almeria, SPAIN

## Abstract

The turning points of housing prices play a significant role in the real estate market and economy. However, because multiple factors impact the market, the prediction of the turning points of housing prices faces significant challenges. To solve this problem, in this study, a historical data-based model that incorporates a multi-population genetic algorithm with elitism into the log-periodic power law model is proposed. This model overcomes the weaknesses of multivariate and univariate methods that it does not require any external factors while achieving excellent interpretations. We applied the model to the case study collected from housing prices in Wuhan, China, from December 2016 to October 2018. To verify its reliability, we compared the results of the proposed model to those of the log-periodic power law model optimized by the standard genetic algorithm and simulated annealing, the results of which indicate that the proposed model performs best in terms of prediction. Efficiently predicting and analyzing the housing prices will help the government promulgate effective policies for regulating the real estate market and protect home buyers.

## Introduction

Real estate not only meets the basic human needs for housing but has also become a way to measure wealth [1]. A large number of countries have an ongoing problem of soaring housing prices and even a bubble, leading to an unhealthy development of the real estate market and difficulty in terms of housing purchasing [2–5]. As the most populous country in the world, this issue is particularly acute in China. The urban housing reforms derived from the last decade have fundamentally changed the Chinese housing market [6,7]. With rapid urbanization in China, the housing market has been stimulated and fueled owing to the continuously increasing demands of residents. In addition, the largest migration from rural regions to urban areas is occurring in China, leading to a rapid increase in the urban population and further sky-rocketing of urban housing prices [8,9]. As a result, there is a conflict between the extensive demand for housing and high housing prices, particularly in metropolitan areas [10,11]. Furthermore, the growth of personal income has been much slower than the increases in housing prices, further aggravating the issue of poor housing affordability [12,13].

As discussed above, low housing prices are disadvantageous for the economy, while high prices make housing unaffordable for residents. In addition, housing prices have an indispensable role in the economy and the stable development of society; consequently, they have attracted the interest of researchers. The prediction of housing prices is an ongoing topic; however, the accurate forecasting of housing prices is challenging owing to multiple factors such as location, public services, and relevant policies. In general, there are two types of predictions in this area. The most popular research direction is in predicting the actual value of a housing price at a specified time in the future [14,15]. Studies in this field have built models with sufficient historical data and influential factors, which can accurately determine the real-time housing prices. The second type is the prediction of the turning points of housing prices because such points are significant signs within a fluctuant time series, upon which the present studies are focused. A turning point refers to a peak of a time series when a long period of the upward trend is stopped, and an evident crash is initiated. Numerous researchers have forecasted the turning points of housing prices using univariate and multivariate methods [16–19]. However, univariate methods are poor at forecasting the downturns or upturns of a time series, whereas multivariate methods rely on the chosen influential factors of the model requiring higher inputs for the selection of independent variables [20].

In this study, the log-periodic power law model optimized through the use of the multi-population genetic algorithm with elitism (LPPL-MPGAWE) is proposed to predict the turning points of housing prices. The LPPL model is applied for forecasting the turning points of housing prices because it introduces no external theory-driven variables and has outstanding interpretability. However, parameter solutions of the LPPL model are quite difficult to achieve because too many parameters need to be determined, and the solution is regarded as a non-deterministic polynomial time-hardness (NP-hard) problem. The complex form of the parameters used and the vast optimal research space are challenged to traditional methods, and the genetic algorithm is therefore considered to be an appropriate choice [21]. Thus, by combining the original genetic algorithm with ideas of multiple populations and a strategy used to retain the best individuals in each generation—elitism, we applied a novel and improved genetic algorithm, MPGAWE, to optimize the parameters of the LPPL model. Based on three cases selected from housing prices in the city of Wuhan, two other widely used heuristic algorithms, the standard genetic algorithm (SGA) [22] and the simulated annealing (SA) algorithm [23], are applied to optimize the LPPL model and are then compared with the proposed method to assess its effectiveness. The results indicate that the LPPL-MPGAWE model is superior to the other two approaches in that it achieves the highest accuracy of the turning points and the lowest sum of the squared residuals (SSR) among the models. Besides, by analyzing the temporal features of housing prices based on government policies, we found that the housing prices at approximately the turning points in Wuhan are affected by such policies to some degree.

The remainder of this paper is organized as follows. Section 2 reviews the related studies on the prediction of housing prices and the solutions to the LPPL model. Section 3 demonstrates the proposed model for turning point forecasting. Section 4 introduces the experimental case of Wuhan and presents the results of the prediction as compared to those of other optimization methods, as well as the results of temporal analysis. Finally, conclusions and limitations of the current study are discussed in Section 5.

## Related work

### Housing price prediction

Housing price prediction as a research area has attracted the attention of numerous researchers. Such predictions are of significant policy interest and are used to forecast the occurrence of peaks and troughs, particularly during periods of economic downturn [24]. The prediction methods used for housing prices can usually be divided into univariate and multivariate methods. The former model types predict the variables using information regarding the previous housing price values and the current state in a time series. In contrast, multivariate methods predict changes to the housing price based on the movement of other explanatory variables.

Numerous examples have proven that univariate autoregressive integrated moving average model (ARIMA) styles perform well at predicting trends and housing price values. Crawford and Fratantoni [16] compared the ability of three types of univariate time series models, namely generalized autoregressive conditional heteroscedastic (GARCH), regime-switching, and ARIMA models, and determined that the latter two outperform GARCH in in-sample and out-of-sample forecasting, respectively. Following this study, Miles [25] employed a generalized autoregressive (GAR) model that performs better than the ARIMA model in markets with high housing-price fluctuations. Vishwakarma [4] proved that the ARIMAX model containing exogenous macroeconomic variables provides better out-of-sample forecasts than other models within the ARIMA family, including ARIMA and ARIMAX-GARCH. Concurrently, the author also verified that the models are only appropriate for short-term predictions. Zietz and Traian [17] used ARIMA-type models, switching regression models, and state-space/structural time series models to forecast the turning point of the US housing market. It is interesting to note that the state-space/structural time series models tend to predict the most accurate results despite not actually fitting the in-sample data.

The other mainstream of the housing price prediction methods use theory-driven multivariate models. Rapach and Strauss [18] found that autoregressive models incorporating economic variables can predict housing prices in the US rather accurately if they do not fluctuate too severely. Croce and Haurin [26] adopted the Granger causality and a Bayesian predictor as the comparison tests and used the good-time-to-buy (GTTB) as an indicator for predicting both the peaks and troughs relatively well. Previous studies also confirmed that a vector autoregressive (VAR), particularly a Bayesian vector autoregressive (BVAR) model, is quite good at predicting the turning points of macroeconomic variables [27,28]. A spatial Bayesian vector autoregressive merged with first-order spatial contiguity and random walk averaging technology has estimated for six metropolitan areas in South Africa by Gupta and Das [29]. Also, GuptaKabundi and Miller [19] employed the ten-variable dynamic stochastic general equilibrium (DSGE) model [30] to forecast the US real estate prices in 2006 successfully and demonstrated that the fundamental economic variables might be improved for predictive purposes. In recent years, DSGE has also been utilized to analyze housing market fluctuations in China [31].

In general, both types of prediction methods have their own advantages and disadvantages. On the one hand, multivariate methods outperform univariate methods at predicting the turning points of housing prices by acquiring a relatively accurate forecast result. On the other hand, if the multivariate methods lack fundamental information, the predicted turning points will be inaccurate. Thus, in this study, the LPPL model is adopted for a turning point prediction consisting of parameters and timestamps without any theory-driven variables. The LPPL model is used specifically to predict the most probable turning points as one of the parameters.

### LPPL model with optimization

The LPPL model was proposed by Johansen et al. and has been used to simulate the growth bubbles in a variety of stock markets, treating the initial market crash as the turning point [32–34]. The premise of the LPPL model is derived from the behavior of the agents in the market. According to the model, traders have only three states: buy, sell, or wait. The actions of traders rely on limited information regarding the decisions of other traders and external influences such as a direct upturn or downturn in the market. A transaction is not an individual event, and each transaction involves two or more traders, indicating that the trading action is irreversible. Based on the above considerations, traders are willing to make trading decisions when profits from rising markets are higher than the losses incurred during a crash, creating a bubble in the market. Thus, the LPPL model is both a statistically physical model and a rational expectation model, and it is also a progressive method for predicting the turning points in the financial market.

Because the LPPL model is derived from the market, numerous researchers have analyzed the bubbles and turning points of housing prices. Zhou and Sornette used the LPPL model to determine whether a bubble exists in the US using quarterly average sale price data. It is notable that the author also showed that the LPPL model was able to successfully predict the critical turning point of the housing price index in UK Halifax [2]. In addition, the LPPL model was applied to estimate the critical time, particularly the crashes of the bubble in Sejong, and in China, Switzerland, and other locations [35–37].

Although the LPPL model is an effective way to predict the turning point of the housing prices, it struggles to obtain the optimal value of the parameters owing to the relatively small and noisy samples used, and numerous parameters need to be determined [38]. The essence of the question is nonlinear and is regarded as an NP-hard problem. Heuristic algorithms are more advantageous than traditional statistical methods to solve this problem, particularly with the advancements in high-speed computing. A Taboo search is commonly used to determine the parameters of the LPPL function from a financial price time series, but it cannot guarantee convergence as a meta-heuristic method [39]. In addition, the SGA and SA methods were also developed to optimize the LPPL model [40–41].

In this study, an improved version of the genetic algorithm called the MPGAWE is applied to search for the best parameters for the LPPL model. Inheriting the advantages of the genetic algorithm, MPGAWE does not rely on the choice of the initial values and is able to obtain global optimization after numerous generations. The MPGAWE guarantees the convergence of the model and avoids the parameters from falling into early maturity owing to the mutation and migration mechanism. This also speeds up the convergence through parallel computing with multiple populations. The details of the LPPL optimized using the MPGAWE are presented in the next section.

## Methodology

In this section, the framework of the LPPL-MPGAWE is presented for forecasting the turning points of housing prices by integrating the LPPL model as the basic means for prediction, and the MPGAWE methods are proposed as an optimized tool for solving the parameters of the LPPL model. This method does not import multiple variables and has perfect interpretability to determine the turning points of housing prices.

### LPPL model and simplification of linear parameters

The LPPL model has been proven to be valuable for detecting financial bubbles and predicting financial crashes as a critical turning point via the fitting of a time series. Because the real estate market involves speculative behaviors, the LPPL model has significant potential to forecast the turning points of housing prices. Specifically, the LPPL model is created as follows [32]:

$$y_{i}=A+B{\left(t_{c}-t_{i}\right)}^{\alpha }+C{\left(t_{c}-t_{i}\right)}^{\alpha }\cos\left(\omega \ln\left(t_{c}-t_{i}\right)+\varphi \right)$$ (1)

Under the current situation, *y*_*i*_ is the real housing price at the time *t*_*i*_, *A*, *B*, *C* are the linear coefficients, α is the exponential growth coefficient, *ω* represents the amplitude of the oscillation, and *φ* is the phase deviation. Most importantly, the model does not require any external theory-driven variables and *t*_*c*_ is the critical time of the crash, which is the most probable prediction time for the turning points of housing prices. By imitating the behavior of traders, the most significant feature of the LPPL is that the amplitude of the oscillation in housing prices decreases and becomes more frequent when *t*_*i*_ is close to *t*_*c*_ as verified from the above equation. The turning points can be predicted by fitting the LPPL model to the historical housing price time series.

However, estimating the parameters of the LPPL model is quite difficult because there are several parameters in the model, including three linear parameters and four nonlinear parameters that need to be optimized. The linear parameters are bound to reduce the computational cost, and they are calculated from the given nonlinear parameters through the ordinary least squares (OLS) method. The original LPPL model in Eq (1) can be compactly rewritten as follows:

$$y_{i}=A+Bf_{i}+Cg_{i}$$ (2)

where

$$f_{i}={\left(t_{c}-t_{i}\right)}^{\alpha }$$ (3)

$$g_{i}={\left(t_{c}-t_{i}\right)}^{\alpha }\cos\left(\omega \ln\left(t_{c}-t_{i}\right)+\varphi \right)$$ (4)

for the given nonlinear *t*_*c*_, *ω*, *φ*, α are linear parameters. In addition, A, B, and C can be obtained through the OLS method as follows:

$$\left(\begin{array}{c} \sum_{i=1}^{N}y_{i}\\ \sum_{i=1}^{N}y_{i}f_{i}\\ \sum_{i=1}^{N}y_{i}g_{i} \end{array}\right)=\left(\begin{array}{ccc} N & \sum_{i=1}^{N}f_{i} & \sum_{i=1}^{N}g_{i}\\ \sum_{i=1}^{N}f_{i} & \sum_{i=1}^{N}{f_{i}}^{2} & \sum_{i=1}^{N}f_{i}g_{i}\\ \sum_{i=1}^{N}g_{i} & \sum_{i=1}^{N}f_{i}g_{i} & \sum_{i=1}^{N}{g_{i}}^{2} \end{array}\right)\left(\begin{array}{c} A\\ B\\ C \end{array}\right)$$ (5)

where N is the number of timestamps. Changing the form of Eq (5) produces the following:

$$\left(\begin{array}{c} A\\ B\\ C \end{array}\right)={\left(\begin{array}{ccc} N & \sum_{i=1}^{N}f_{i} & \sum_{i=1}^{N}g_{i}\\ \sum_{i=1}^{N}f_{i} & \sum_{i=1}^{N}{f_{i}}^{2} & \sum_{i=1}^{N}f_{i}g_{i}\\ \sum_{i=1}^{N}g_{i} & \sum_{i=1}^{N}f_{i}g_{i} & \sum_{i=1}^{N}{g_{i}}^{2} \end{array}\right)}^{-1}\left(\begin{array}{c} \sum_{i=1}^{N}y_{i}\\ \sum_{i=1}^{N}y_{i}f_{i}\\ \sum_{i=1}^{N}y_{i}g_{i} \end{array}\right)$$ (6)

After reaching this step, only four free parameters remain to be determined. However, the optimization of the best value of the nonlinear parameters is challenging owing to its complexity. Based on previous research, MPGAWE derived from the SGA is applied to the solution. The next section provides details of the MPGAWE model.

### MPGAWE for non-linear parameter optimization

As discussed above, the solution of the nonlinear parameters to the LPPL model is an NP-hard problem. As far as can be determined, the genetic algorithm is one of the most popular methods used to solve the NP-hard problem. The MPGAWE model surpasses the ordinary genetic algorithm by establishing a multi-population framework and introducing the migration mechanism and elitism, which increases the precision of the prediction and the speed of the convergence.

The MPGAWE procedure can be divided into representation, initialization, selection, crossover, mutation, reinsertion, and migration operators.

#### (1) Representation

**Fig 1:**
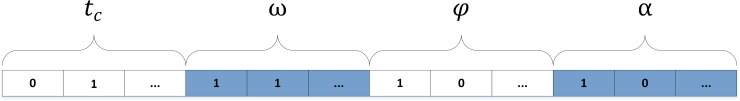
Representation for an individual.

In this study, each individual consists of four nonlinear parameters of the LPPL model (*t*_*c*_, *ω*, *φ*, α). Binary coding is used for each parameter because it is a user-friendly coding technique that can alter the precision with a suitable length. The original parameters are encoded into binary genes and chromosomes are formed with the genes stacked up such that a fixed number of genes represent the actual value of the parameter and all genes in the individual represent a series of nonlinear parameters. An example of an individual is shown in Fig 1.

#### (2) Fitness

The fitness is of significant importance in the genetic algorithm, reflecting the performance of each individual. In this study, the fitness value of an individual is simply determined as the sum of the squared residuals (SSR). This indicator is widely used to assess the error between the estimated value and the true value. The fitness in the MPGAWE is described as follows:

$$f_{m,n}=\sum_{i=1}^{N}{\left\{y_{i}-A-B{\left({t_{c}}^{m,n}-t_{i}\right)}^{\alpha^{m,n}}-C{\left({t_{c}}^{m,n}-t_{i}\right)}^{\alpha^{m,n}}cos(\omega^{m,n}\ln\left({t_{c}}^{m,n}-t_{i}\right)+\varphi^{m,n})\right\}}^{2}$$ (7)

where *f*_*m*,*n*_, *t*_*c*_^*m*,*n*^, *α*^*m*,*n*^, *ω*^*m*,*n*^, and *φ*^*m*,*n*^ are the fitness values and four nonlinear parameters of the LPPL corresponding to the n-th individual in the m-th population. Thus, for each generation, the fitness of every individual can be calculated using the given nonlinear parameters.

#### (3) Initialization

It has already been proven that the results from the genetic algorithm do not significantly rely on the initialization; thus, to simplify the method, a random initialization is adopted in this study. During the initialization step, all populations with a certain number of individuals are generated by randomly selecting 0 or 1 for each gene. The number of genes can be computed as follows:

$$N_{gene}=N_{p}\times S_{p}\times L_{c}$$ (8)

where *N*_*p*_ is the number of population, *S*_*p*_ is the size of population and *L*_*c*_ is the length of the chromosomes.

#### (4) Selection

The selection operator depends on the fitness values of the individuals, and there are two parts of individuals to be chosen with the same individual number. The first part is called the *elites* with a fixed small number, *eliteSize*, made up of the best individuals within the population, which proceed straight to the next generation. With the *elitism* mechanism, the best individuals in each generation are protected to guarantee that the fitness of the next generation will not be at risk. The second part is the basis for subsequent operations; and is selected via classical roulette selection with the following probability:

$$P_{i}=\frac{\frac{1}{f_{i}}}{\sum_{j=1}^{N}\frac{1}{f_{j}}}$$ (9)

where *P*_*i*_ and *f*_*i*_ represent the probability to be selected and the fitness value of individual *i*, respectively.

#### (5) Crossover

**Fig 2:**
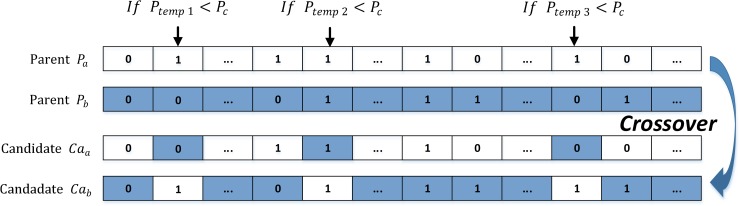
Uniform crossover based on selected parents.

*Uniform crossover* is used in this step. For the ordinary selected individuals, we pair them two by two as a set of parents, meaning that *eliteSize* should be designed to keep the residual individuals even. With the given probabilities *P*_*c*_ in different populations and a random value, each pair of genes from the selected parents can be selected for swapping (see Fig 2).

#### (6) Mutation

**Fig 3:**
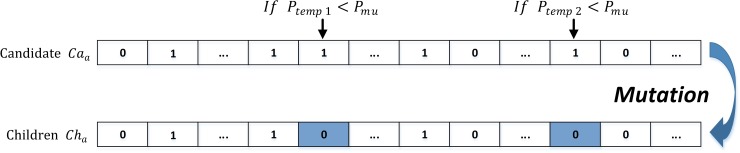
Mutation operator with a small probability.

Following the laws of natural reproduction, we design the mutation operator using a random selection method. By assigning a small mutation probability *P*_*mu*_, the gene of the individual is selected for the mutation if a number *P*_*temp*_ randomly chosen between 0 and 1, is smaller than *P*_*mu*_. The offspring are generated by mutating the genes through 0 to 1 or through 1 to 0 (see Fig 3).

#### (7) Reinsertion

At the beginning of the reproduction, the selected individuals are divided into *elites* and others. Thus, we reinsert the *elites* into the candidates generated by the selection, crossover, and mutation to form a new generation. Simultaneously, the fitness values of individuals need to be recomputed, and the individuals with the best performance in each population and all chromosomes are recorded.

#### (8) Migration

**Fig 4:**
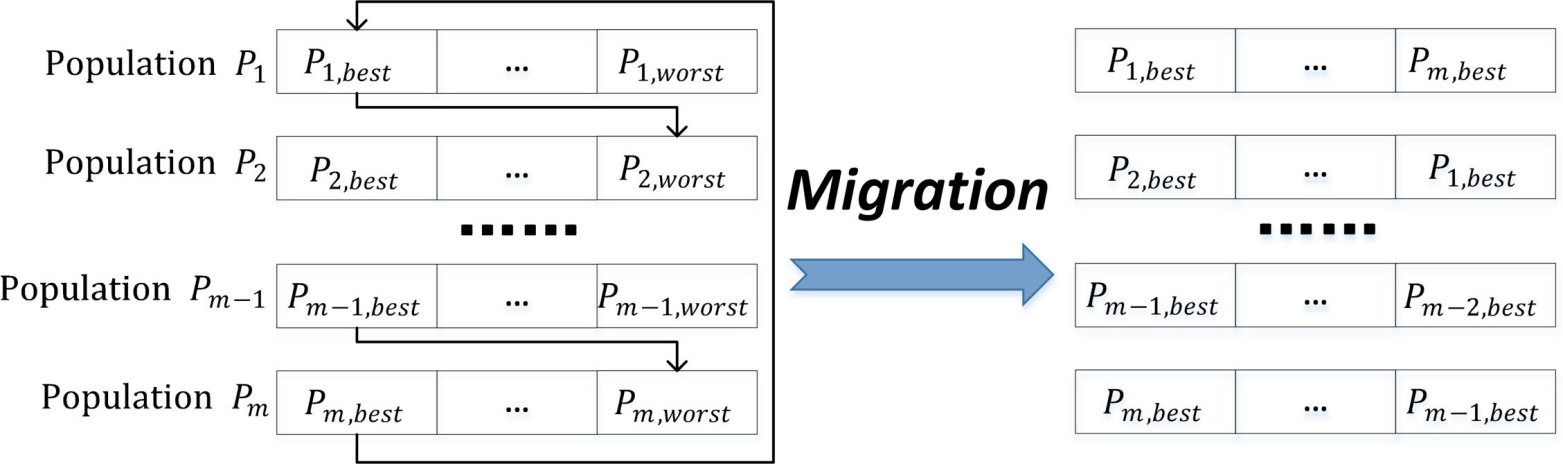
Best individuals migration between populations.

Migration is the most significant operator in the MPGA in comparison to the SGA. At the end of each epoch, the chromosome i with the smallest fitness value in the m-th population replaces the chromosome j with the largest fitness value in the m+1-th population. As before, the individual with the best performance will be repeatedly recorded (see Fig 4).

Notably, owing to such numerous local optima in the fitness function, the number of generations needs to be quite large to acquire a reliable result. After several iterations, the value of the nonlinear parameters will converge and be determined, from which the entire LPPL model can be established. The pseudo-code of the MPGAWE used to optimize the LPPL model is as follows:

Algorithm 1. MPGAWE for optimizing the LPPL model

    popNum—desired number of populations

    popSize—desired population size

    eliteSize—desired number of elite individuals

    maxGen—maximum generations (relatively large)

    *p*_*mu*_ – probability of mutation (relatively small)

    *P*—the populations up to the generation

    *fit–*the fitness values of each population = ∅

    *bestFit–*the lowest fitness value among the candidates = ∅

    *bestInd–*the best individual among the candidates = ∅

    *P* = *Initialization* (*popNum*,*popSize*)

    **do**

        **for** each population *m*∈*popNum* **do**

            **for** each individual *n*∈*popSize* **do**

                *fit*_*m*,*n*_ = *AccessFitness*(*P*_*m*,*n*_)

                **if *bestFit* = ∅** or *fit*_*m*,*n*_<*bestFit*:

                    *bestFit* = *fit*_*m*,*n*_ and *bestInd* = *P*_*m*,*n*_

                **end if**

            **end for**

        **end for**

        **for** each population *m*∈*popNum* **do**

            *Q* = the *eliteSize* fittest individuals in *P*_*m*_

            **for (*popSize*−*eliteSize*)/2** times **do**

                Parent *P*_*a*_ = *roulette Selection*(*P*_*m*_)

                Parent *P*_*b*_ = *roulette Selection*(*P*_*m*_)

                Candidate *Ca*_*a*_,*Ca*_*b*_ = *single-point Crossover*(*copy*(*P*_*a*_),copy(*P*_*b*_))

                Children *Ch*_*a*_,*Ch*_*b*_ = *Mutation*(*copy*(*Ca*_*a*_),*copy*(*Ca*_*b*_),*p*_*mu*_)

                *Q* = *Q*∪{*Ch*_*a*_,*Ch*_*b*_}

            **end for**

        **end for**

        the best individual in *P*_1_ replaces the worst individual in *P*_*final*_

        **for** each population *m*∈*popNum* **do**

            the best individual in *P*_*m*+1_ replaces the worst individual in *P*_*m*_

        **end for**

        *P = Q*

    **until** epoch reach *maxGen*

    **return** *bestFit*, *bestInd*

### LPPL-MPGAWE for prediction and assessment

**Fig 5:**
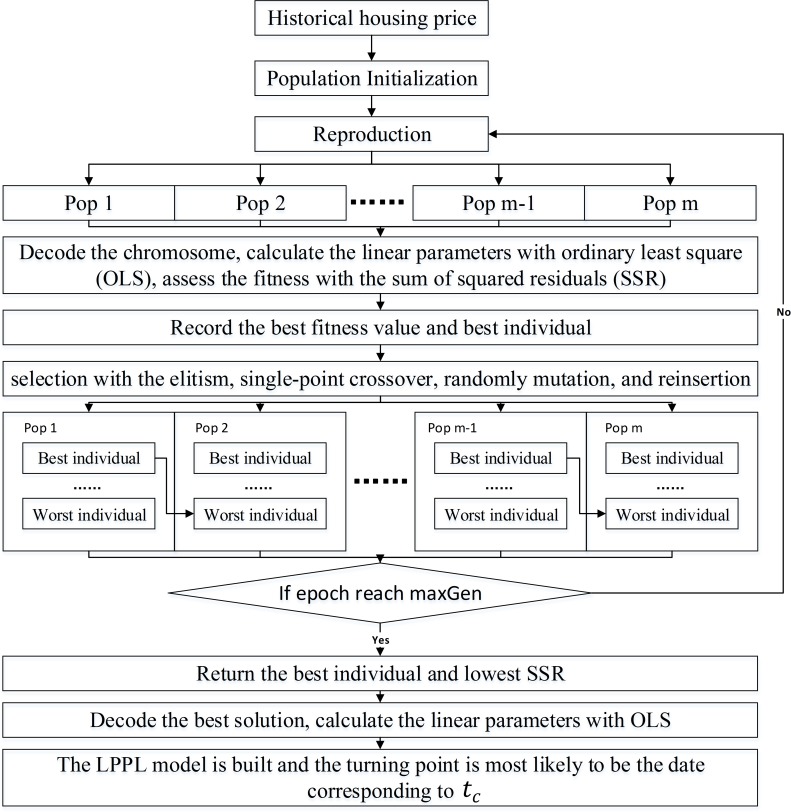
Flowchart of LPPL-MPGAWE.

Similar to the operators described above, the MPGAWE is used to optimize the nonlinear parameters of the LPPL model. At the same time, the remaining parameters can be determined through the given nonlinear parameters and the least squares method, which results in the entire LPPL model being able to predict the most probable turning points of housing prices. The overall method of the framework can be seen in Fig 5.

By referring to Geraskin and Fantazzini [38], the thresholds and values of the parameters of the LPPL and MPGAWE used in this study are as shown in Table 1.

**Table 1:** Parameter values and thresholds used in the experiments.

Parameter	Threshold/Value
*t*_*c*_	(0,60] (two months after the last historical day)
*ω*	[6,15]
*φ*	(0,2 π)
α	[0.1,0.9]
*popNum*	5
*popSize*	20
*eliteSize*	4
*maxGen*	1000
*p*_*c*_	[0.4,0.6]
*p*_*m*_	[0.05,0.1]

Finally, we assess the reliability of the model using the parametric detrending approach suggested by Zhou and Sornette [42]. This method is divided into two steps where we construct the residual (periodic oscillations of the LPPL model) in the form of *res*(*t*) = [*y*(*t*)−*A*]/(*t*_*c*_−*t*)^*α*^ to detrend *y*(*t*), and the Lomb periodograms of the residual are then calculated. The results of the Lomb periodograms indicate the frequency *f* with the best fit to the residual. The model exhibits a great effect when *f*≈*ω*/2*π*, where *ω* is the angle-frequency of the LPPL model.

## Materials

### Case study: Wuhan

Wuhan, the capital city of Hubei province located at the junction of the Yangtze and Han Rivers, is one of the most important transportation hubs and economic centers in China. The city is composed of seven central districts and six distant districts with a land area size of 8,569.15 km^2^ and a resident population of 10.89 million (as of 2017). With an improvement in the living standards of its residents, rising housing prices in Wuhan have attracted significant attention. In terms of time, there is a similar upward trend of the housing prices in Chinese metropolitan areas from 2016 to 2018, and some turning points occurred owing to the market effect and management by policies. Taking Beijing as an example, the average housing price was 39,423 Yuan/km^2^ in January 2016 and rose to 62,957 Yuan/km^2^ in January 2018, with housing prices barely rising during the second half of 2017. Moreover, with using data from December 2016 to September 2018 supported by the Wuhan Natural Resources and Planning Information Center, the fine-grained housing price dataset in Wuhan can be acquired for analysis with a high temporary and spatial resolution. To summarize, the dataset of housing prices in Wuhan city from December 2016 to September 2018 offers a perfect case study for this research.

### Housing price dataset and preprocess

There are two types of datasets used to verify the reliability of the model, t. A total of 30,218 real estate records were collected from the Wuhan Natural Resources and Planning Information Center. The data contain detailed real estate information, including the unit number, location, ownership, and transfer time of the property. However, the transaction values in some records are missing. To solve this problem, we collected housing price data from the Anjuke website (https://wuhan.anjuke.com), which includes information from 4,702 residential districts around the Wuhan area (shown in S1 Table).

During the preprocessing stage, the housing price data were combined with real estate information through place name address matching and spatial location matching, which completely fills in the gaps in the real estate records. Then, the data were washed by deleting the redundant records and removing the holiday statistics because the records on weekends are far fewer in number than those on the weekdays. Anomaly detection according to the three-sigma principle is conducted, and the abnormal records are removed. We use a 20-day moving time window to calculate the average values of the data as a sample because the housing prices should change smoothly within a few days, avoiding drastic fluctuations caused by noise and the differences from individual transaction records. For analysis at different spatial scales, the data are divided based on the administrative district and the time series created for the different areas.

### Selection of turning points and training samples

**Fig 6:**
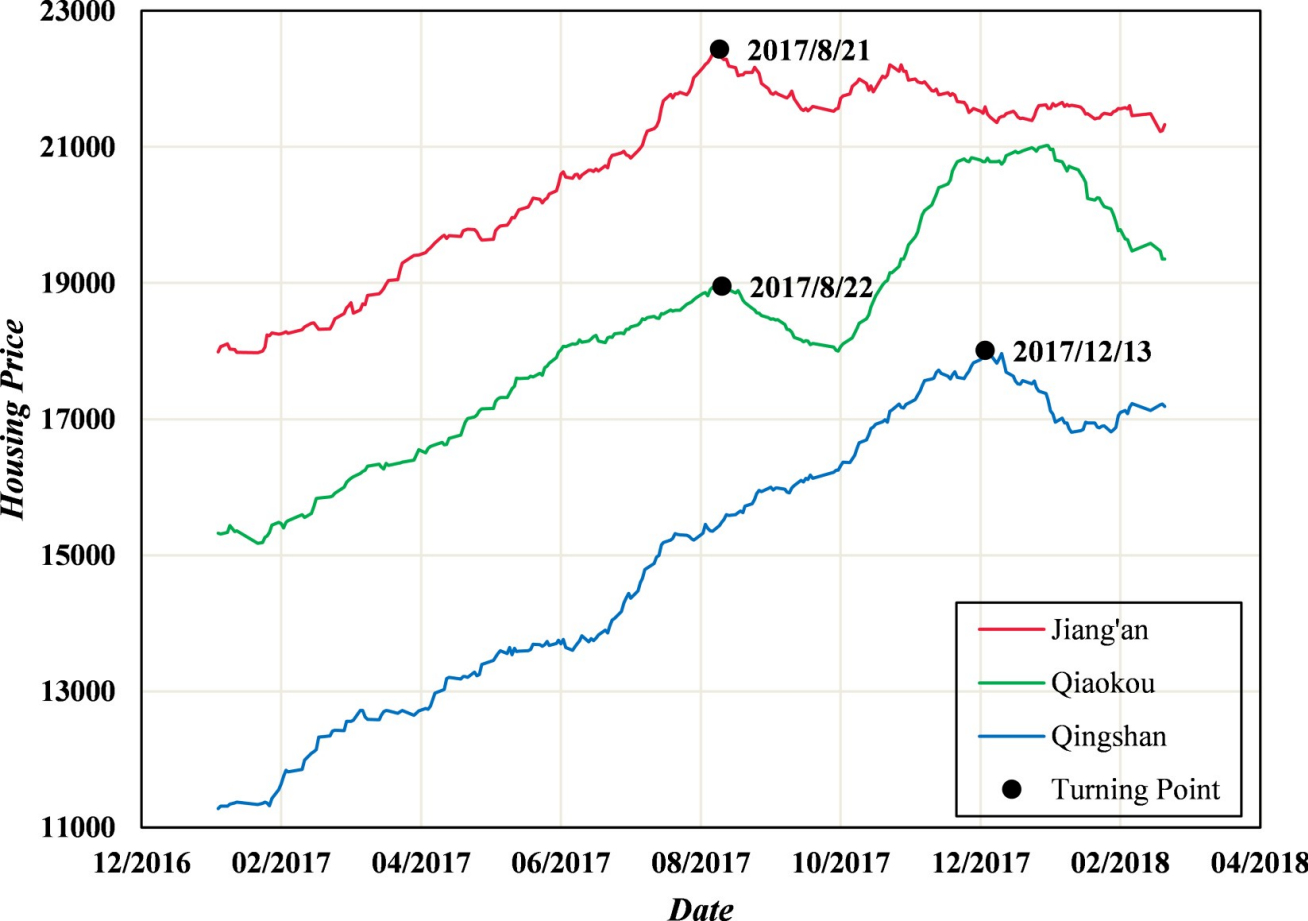
Original time series of housing prices in the study areas.

Based on the raw dataset, the turning points in the time series need to be determined, and the corresponding samples need to be selected for training. A turning point is the critical time of a peak in a bubble at which a crash begins. Unlike in the stock market, the fluctuations in the housing prices are always gentle and smooth. Thus, when considering the characteristics of the housing price dataset and the reference standards in [43] and [44], we identified a turning point as follows: (1) 180 weekdays prior to the turning point for which there is no housing price higher than the peak, and (2) a drop in housing prices of 4% within a period of 45 weekdays after the turning point. Based on these criteria, three turning points (peaks) on August 21 and 22 and December 13, 2017 in the Jiang’an (case 1), Qiaokou (case 2), and Qingshan (case 3) districts are identified respectively (as shown in Fig 6). The data on the housing prices for the experiment are presented in S2–S4 Tables, which are clipped from January 2017 to February 2018 because there no significant turning points in the remaining part. The selection of the training samples needs to be noted, we designed the time window for the training samples by referring to previous studies [43, 45–46] when considering that the period of the data is limited: (1) Because the time of the previous crash is difficult to acquire, the lowest point since the start of the dataset is regard as the beginning of the time window; (2) To reserve a period of data for prediction, the last day of the month two months prior to the turning point is regarded as the endpoint of the time window (e.g., the turning point is May 15 and the endpoint is March 31). (3) It was confirmed that the period of the training set is longer than 120 weekdays, and there is no sharp downturn in this set. Finally, data from January 13 to June 30, 2017 in case 1, February 4 to June 30, 2017 in case 2, and January 17 to October 30, 2017 in case 3 were chosen for the modeling in the three districts, respectively.

## Results and analysis

This section presents an evaluation of the housing prices in Wuhan during December 2016 and October 2018. Based on the three cases selected from the overall dataset, we demonstrate the reliability and effectiveness of the proposed model through a comparison with the LPPL model improved using the two other heuristic algorithms.

### Results of LPPL-MPGAWE

**Fig 7:**
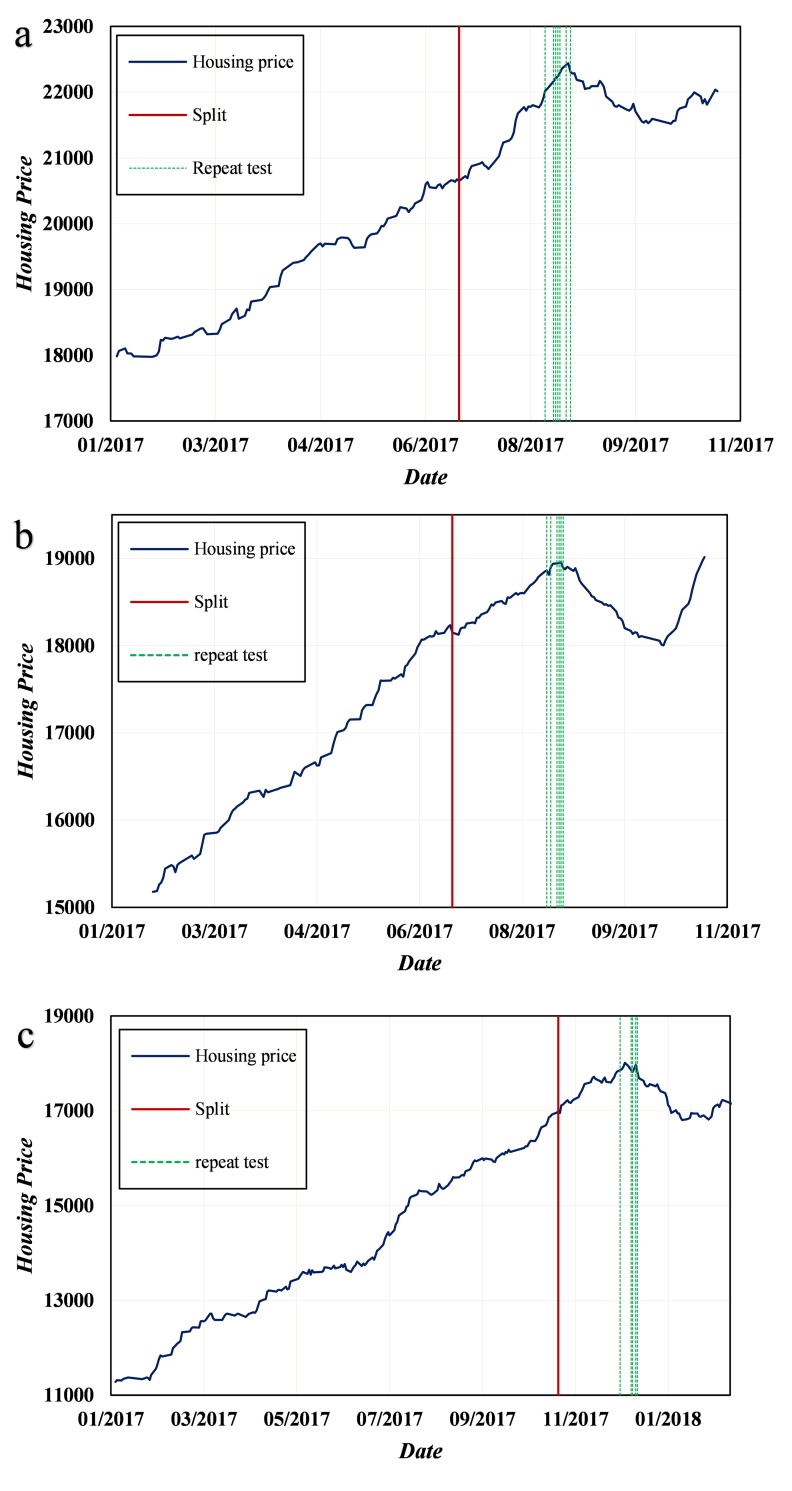
Repeated tests of LPPL-MPGAWE for three cases. (a) Results for the Jiang’an district (case 1). (b) Results for the Qiaokou district (case 2). (c) Results for the Qingshan district (case 3).

Because the fitness function of the MPGAWE algorithm has multiple location minimums, we repeat the models ten times for the data of each region and select the parameters with the lowest costs. Fig 7 shows the results of the LPPL-MPGAWE model with data from the three districts. The red lines are split to represent the splitting of the sample data, and the green lines are the timestamps of the turning points for each repeated test. In case 1, the turning point was focused on August 17, 2017, and in case 2 and case 3, the turning point was concentrated on August 20 and December 20, 2017, respectively. Clearly, although the LPPL-MPGAWE method is built using no theory-driven factors, the results converge at the real turning points with slight fluctuations, and the model is stable.

The parameters of the LPPL model with the lowest SSR values are shown in Table 2. There is a small gap between *ω* calculated by the Lomb periodograms and *ω* calculated using the MPGAWE, which is caused by the short sampling period, and fewer complete fluctuation cycles are detected. The results are consistent with the conclusion that the parameter is most readily optimized when *ω*≈6.36±1.56, and another angle-frequency occurs at approximately *ω*≈11.5 for a few cases, presented as the “second harmonics”, which was proven through numerous experiments conducted by Johansen [47].

**Table 2:** Parameters of the LPPL model fits for three cases.

Case	A	B	C	*t*_*c*_	*ω*	*φ*	α	*ω*_*Lomb*_	*t*_*real*_
1	22036.18	-33.18	-1.64	2017-08-17	11.3	5.12	0.89	12.0	2017-08-21
2	19723.53	-38.87	1.43	2017-08-23	7.5	5.26	0.88	6.5	2017-08-22
3	18445.05	-38.56	-1.90	2017-12-18	7.4	5.20	0.90	6.5	2017-12-13

**Fig 8:**
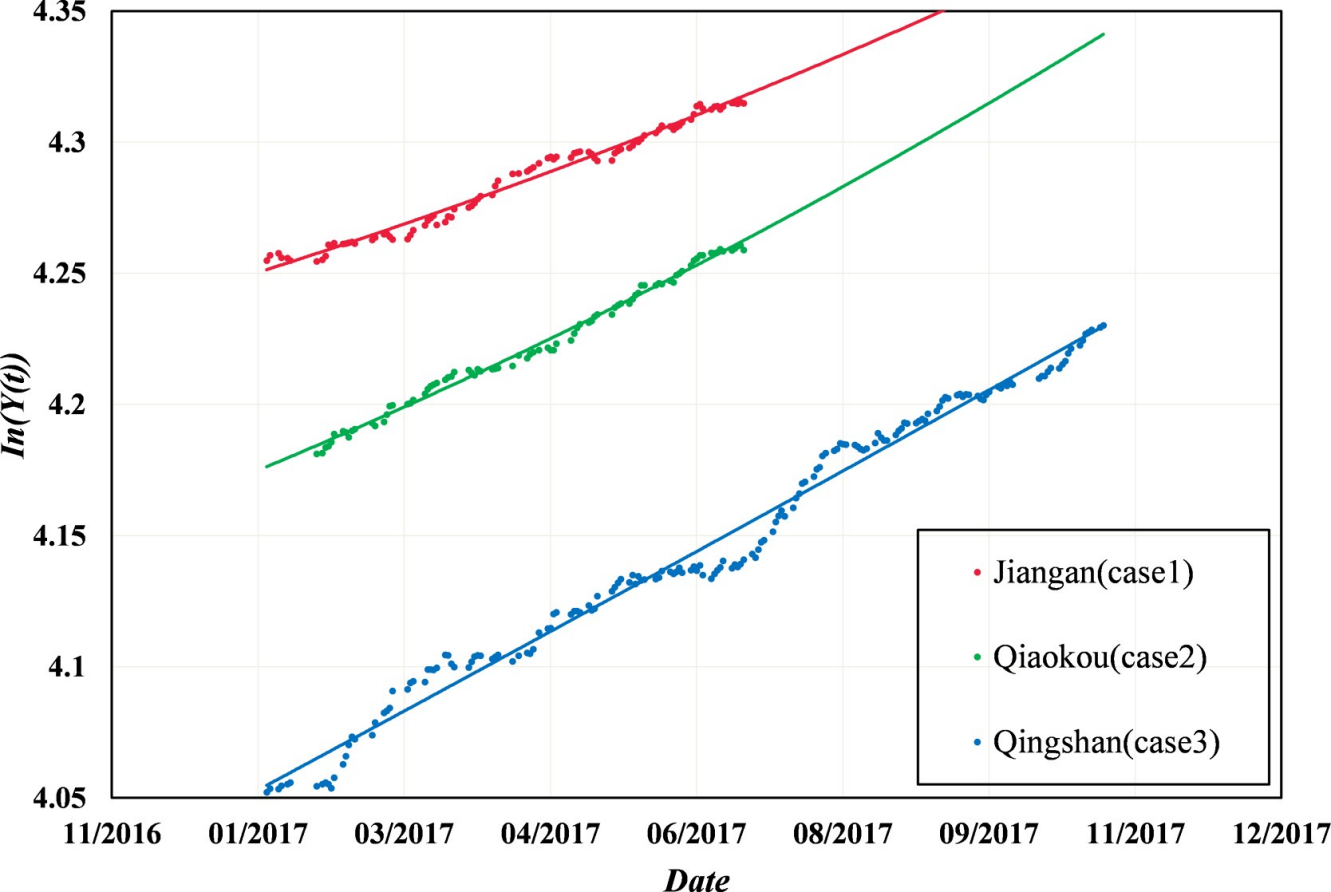
Exponential growth analysis with three case samples.

The α parameter is used to describe a faster-than-exponential growth of the housing price, and it is worth noting that α obtained from the method nearly reached the upper limit (0.9) for the three cases, which is almost pathologic. Thus, an exponential growth analysis is applied to fit the samples for all cases. In general, a faster-than-exponential growth is identified by a strong upward trend in the curves, whereas in Fig 8, the curves tend to be straight lines with a slight upward curvature. Owing to the characteristics of the faster-than-exponential growth, which are difficult to identify, the model nearly degenerates into a linear form without exponential growth, and the α parameter also nearly reaches the upper limit.

### Comparison among LPPL-MPGAWE, LPPL-SGA, and LPPL-SA methods

**Fig 9:**
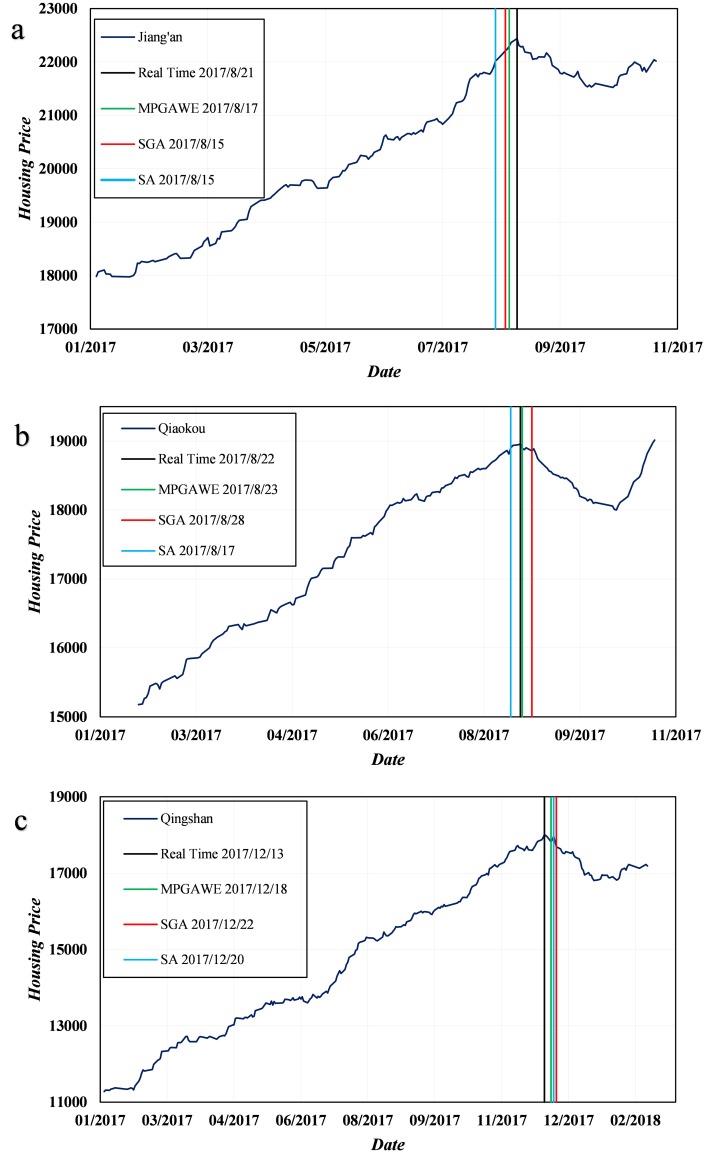
Results of the turning point predictions with different algorithms for the three cases. (a) Comparison of the three algorithms with the samples from Jiang’an district (case 1). (b) Comparison of the three algorithms with the samples from the Qiaokou district (case 2). (c) Comparison of the three algorithms with the samples from the Qingshan district (case 3).

To further demonstrate the ability of LPPL-MPGAWE for predicting the turning points of the housing prices, the method is compared with the LPPL improved by the SA and SGA methods, and each type of model is established based on the optimal parameters for the three cases, respectively. The results of the forecasting using the three methods are shown in Fig 9. In the figure, the blue curve is the original time series of the housing prices, and the vertical black line indicates the real date of the turning point. The green lines indicate the turning points as predicted by the LPPL model improved with the MPGAWE, whereas the red lines and blue lines represent the results of the LPPL model improved with the SGA and SA, respectively. The results indicate that in all three cases, the predictions generated by LPPL-MPGAWE are closest to the actual dates of the turning points among the three methods. Notably, the results of LPPL-SA deviate relatively from the real value of the turning point in case 1 (beyond one week). Although the LPPL-SGA has a great ability to forecast, it is not a patch on the LPPL-MPGAWE because it is prone to local optimization, for which a large number of tests need to be carried out before obtaining a good result. Table 3 shows the values of the SSR of all algorithms for the three cases and suggests that LPPL-MPGAWE performs best at predictions with the lowest cost under almost every scenario. To summarize, MPGAWE is superior to the other two heuristic algorithms when optimizing the parameters of the LPPL model and is available for turning point predictions.

**Table 3:** Comparison of the sum of the squared residuals (SSR) among the three algorithms.

Case	MPGAWE	SGA	SA
1	434294.8	418042.5	440603.5
2	309074.5	310687.7	415107.5
3	4169220.7	4186114.7	5582987.5

### Temporal analysis with policies

Overall housing prices in Wuhan have been rising consistently since October 2016, with some areas showing a downtrend. As described above, there were two major turning points in August 2017 and December 2017 in some districts in Wuhan (as shown in Fig 6). To a certain degree, the housing price fluctuations are driven by policies macroscopically regulated by the government. Therefore, two significant points need to be noted at the time of the turning points. From the end of 2016 to April of 2017, the average mortgage rate in Wuhan was below 4.5%. With the government controlling the rocketing housing prices economically, the average mortgage rate continued to rise, and a qualitative change took place in July 2017 when the rate exceeded 5%. As a result, the high average mortgage rate limited the consumption-ability of home buyers and inflated the housing prices observably within in a few months, generating the turning points in August 2017. More importantly, on October 18, 2017, the 19th CPC National Congress clearly stated that “Houses are used for living, but not for speculation”, after which, the government departments at all levels positively responded to this declaration. Specifically, the Department of Housing and Urban-Rural Development of Hubei Province introduced a policy of “Notice on the implementation of real estate market regulation and control due to urban conditions” on November 20, emphasizing rational management of the real estate market to deal with price gouging and deliberate housing market types strictly. Under the high pressure of such regulations by the government, the rising trend in the housing prices was restrained, leading to a turning point in December 2017. In general, policies of regulation and management are relatively effective for controlling housing prices in a period of time, resulting in the stable development of the real estate market. However, because the impacts of such policies are time-sensitive and the comprehensive market reasons for rising housing prices remain, the housing prices in some areas have risen again.

## Conclusion and discussion

In this study, a novel framework called LPPL-MPGAWE was applied by incorporating a financial expectation model and a reliable heuristic algorithm to forecast the turning points of the real estate market accurately. The study results show an improvement with the proposed method in two aspects. (1) By combining the mechanism of multiple populations and elitism with a genetic algorithm, we developed the MPGAWE model to determine the optimal nonlinear parameters of the LPPL model. The MPGAWE is effective for dealing with complex nonlinear problems, and it has advantages that the result does not rely on the initial values, and the convergence of the optimizing parameters is guaranteed. (2) The LPPL model was built through historical information without any external theory-driven variables for predicting the turning points of housing prices, and achieves a high interpretability of the turning point because the turning point corresponds to the parameter in the model.

With the case study conducted in Wuhan city, the LPPL-MPGAWE model performs well in the forecasting of three cases with a lower SSR than the LPPL-SA and LPPL-SGA models. The proposed method also has a significant potential of predicting the turning points of housing prices in other cities that have a similar trending fluctuation in the time series. Besides, the LPPL model is also a state-of-the-art method applicable to the forecasting of the time series with soaring bubbles in other fields, such as the stock market, the oil market, and commodity prices. In the application of these fields, the LPPL improved with MPGAWE can obtain precise optimal parameters and increase the accuracy of the prediction.

Although the LPPL-MPGAWE model seems suitable for prediction, it also has some obvious limitations. The simple form of the LPPL model has difficulty describing the real estate market in China because it only considers stimulating the trader behavior in the market, which ignores the influence of government policies. Although the LPPL model has been proven to be robust using sampling data with different time windows [42], there are still certain additional data requirements when the model is applied, namely, the relatively long period of the samples for modeling, which means it is quite difficult to achieve a real-time prediction. The parameters of the LPPL-MPGAWE model have slight gaps with the results of the Lomb periodograms, which are caused by insufficient sample data, indicating that the period of the sample data also influences the accuracy of the prediction.

In future studies, we will consider the impact of policies for improving the model. We will further employ the model to forecast the turning points of housing prices in other cities and even other markets to verify its broad applicability. Finally, the method can be used to help local governments manage the real estate markets, promoting the steady development of the real estate market. At the same time, home buyers will be able to acquire valuable advice through the proposed method.

## Supporting information

S1 Table Raw housing price data in Wuhan from the website https://wuhan.anjuke.com. (XLSX)

S2 Table Data of housing prices in Jiang’an district for case 1. (XLSX)

S3 Table Data of housing prices in Qiaokou district for case 2. (XLSX)

S4 Table Data of housing prices in Qingshan district for case 3. (XLSX)
